# Supplementary material for: Reorganisation of Cortico-Hippocampal White Matter Pathways in Healthy Ageing: Evidence for Paradoxical Shifts in Structural Connectivity
Source: bioRxiv. 2025 Dec 2:2025.11.28.691222. Preprint. [Version 1] doi: 10.64898/2025.11.28.691222 (PMC12822711; doi:10.64898/2025.11.28.691222)
Supplement: Supplementary file 1 [file media-1.pdf]

Supplementary Table 1. List of abbreviations for the Top 20 most highly connected cortical areas. Listed by rank order of connectivity strength in the younger group.

**Cortical Area**	**Name**	**Brain Region**
EC	Entorhinal Cortex	Medial Temporal Cortex
PeEC	Perirhinal / Ectorhinal Cortex	Medial Temporal Cortex
V1	Primary Visual Cortex	Early Visual Cortex (Occipital)
V2	Second Visual Area	Early Visual Cortex (Occipital)
V3	Third Visual Area	Early Visual Cortex (Occipital)
TGv	Area TG Ventral	Lateral Temporal Cortex / Temporal Pole
POS2	Parieto-Occipital Sulcus Area 2	Medial Parietal Cortex
TGd	Area TG dorsal	Lateral Temporal Cortex / Temporal Pole
POS1	Parieto-Occipital Sulcus Area 1	Medial Parietal Cortex
PHA1	Parahippocampal Area 1	Medial Temporal Cortex
RSC	Retrosplenial Complex	Medial Parietal Cortex
TF	Area TF	Lateral Temporal Cortex
PHA2	Parahippocampal Area 2	Medial Temporal Cortex
V3A	Area V3A	Dorsal Stream Visual Cortex
V6	Sixth Visual Area	Dorsal Stream Visual Cortex
DVT	Dorsal Transitional Visual Area	Medial Parietal Cortex
ProS	ProStriate Area	Medial Parietal Cortex
PHA3	Parahippocampal Area 3	Medial Temporal Cortex
Pir	Pirform Cortex	Frontal Opercular Cortex
V4	Fourth Visual Area	Early Visual Cortex (Occipital)
